# Comparative Evaluation of Annual and Comprehensive Maintenance Contracts for Reducing Medical Equipment Downtime in a Tertiary Care Hospital: An Observational Audit

**DOI:** 10.7759/cureus.113345

**Published:** 2026-07-25

**Authors:** Shruti Mahto, Shailesh Tripathi, Vanini, Pushpanjali, Anit Kujur

**Affiliations:** 1 Department of Hospital Administration, Rajendra Institute of Medical Sciences, Ranchi, IND; 2 Department of Trauma Pathology, Rajendra Institute of Medical Sciences, Ranchi, IND; 3 Department of Community Medicine, Rajendra Institute of Medical Sciences, Ranchi, IND

**Keywords:** annual maintenance contract, comprehensive maintenance contract, equipment downtime, healthcare technology management, tertiary care hospital

## Abstract

Background: Medical equipment availability is essential for safe, continuous, and efficient care delivery in tertiary care hospitals. Downtime of diagnostic, operative, monitoring, and life-support equipment can disrupt clinical services, delay procedures, and compromise patient safety. Maintenance models such as Annual Maintenance Contracts (AMC), Comprehensive Maintenance Contracts (CMC), warranty coverage, and in-house repair systems differ in scope, cost predictability, and risk protection.

Objective: This study aimed to comparatively evaluate maintenance-contract coverage and equipment functionality patterns associated with AMC, CMC, warranty, and no active contract status in a tertiary care hospital. It also assessed departmental vulnerability and maintenance gaps to support risk-based planning and clinical service continuity.

Methodology: A cross-sectional observational audit was conducted using biomedical equipment inventory records from selected clinical and diagnostic departments. Equipment entries were assessed for department, contract status, warranty status, and functional condition. Contract coverage was categorised as protected, partially protected, or unprotected, and descriptive analysis was used to identify departmental maintenance gaps and non-functional equipment clusters.

Results: The audit demonstrated that the absence of active maintenance coverage was associated with greater vulnerability to equipment non-functionality, particularly among high-value and clinically critical devices. CMC offered broader protection through preventive service, corrective repair, and spare-part inclusion.

Conclusion: Risk-based maintenance planning is required, with CMC prioritised for critical equipment and structured AMC or in-house systems used for lower-risk assets.

## Introduction

Medical equipment is central to the delivery of safe, timely, and effective care in tertiary care hospitals [1]. Diagnostic imaging units, laboratory analysers, anaesthesia workstations, endoscopic systems, surgical energy platforms, intensive care ventilators, and patient monitoring devices support clinical decision-making across emergency, operative, inpatient, and critical care settings [2]. Any interruption in equipment availability can delay diagnosis, postpone procedures, disrupt patient flow, increase referral burden, and compromise continuity of care [3]. In high-volume tertiary hospitals, equipment downtime is not only a technical event; it is a patient-safety and health-system performance concern [4]. Maintenance planning is a core component of healthcare technology management [1]. Annual Maintenance Contracts (AMC), Comprehensive Maintenance Contracts (CMC), manufacturer warranty coverage, in-house biomedical engineering support, and pay-per-repair arrangements represent common strategies used to maintain equipment functionality [5]. An AMC usually includes scheduled preventive maintenance and breakdown service support, with spare parts billed separately [6]. A CMC generally includes preventive maintenance, corrective maintenance, labour, travel, and spare parts within a single contract, offering broader financial predictability and stronger vendor accountability [4]. Warranty coverage provides manufacturer-supported repair during the initial post-installation period. Equipment without active warranty or maintenance coverage relies primarily on institutional response capacity, local vendor availability, or ad hoc repair expenditures [7].

Maintenance contracts have more significance than just the type of contract. Actual downtime risk may change depending on equipment criticality, age, utilisation, availability of backups, cost of the spare parts, vendor response time and biomedical engineering capacity [8]. Low-cost and easily replaceable equipment can be covered by a simpler policy than life-support and high-value operative equipment [9]. The choice of a contract in a tertiary care setting should consider clinical risk, reliance on services, and impact on operations, not initial cost [3]. Existing evidence indicates that full maintenance contracts can increase equipment availability and minimise downtime over equipment that does not have active service coverage [10]. Existing studies also indicate that warranty-like or comprehensive support can produce higher availability than informal or reactive maintenance models [11]. Even with this, there are still numerous hospitals that experience gaps after warranty expiration, lack preventive maintenance records, have delayed contract renewals, and have not adequately tracked non-functional equipment by department. There is not yet enough evidence of the relationship between AMC, CMC, the warranty status and the absence of active contracts and the actual functioning patterns in clinical departments in tertiary care hospitals.

Another discrepancy is between the literature-derived downtime estimates and institutional biomedical inventory data. There are numerous published discussions on the theory of cost comparison or general equipment management concepts, but most concentrate on maintenance contracts. Only a limited number of hospital-based evaluations evaluate department-wise contract coverage, functional status, high-risk equipment clusters and implications for service continuity within a single tertiary care facility. This information is essential for quality improvement, preparedness for accreditation, capital planning and patient-safety governance.

Objectives of the study

This study aimed to comparatively evaluate maintenance-contract coverage and medical equipment functionality patterns across AMC, CMC, warranty, and no active contract categories in a tertiary care hospital. It also assessed departmental vulnerability, non-functional equipment burden, and maintenance gaps to support risk-based maintenance planning and clinical service continuity. The comparison was descriptive and based on observed inventory patterns; no inferential or causal comparison of AMC and CMC effectiveness was performed.

## Materials and methods

Study design and setting

A cross-sectional observational audit design was used to assess the relationship between medical equipment maintenance coverage and functional availability in a tertiary care teaching hospital. The audit focused on biomedical equipment recorded in selected clinical and diagnostic departments, with particular attention to service-continuity risk, departmental maintenance coverage, and the burden of non-functional equipment. The selected departments were included based on the availability of equipment-level inventory records containing sufficient information on equipment identity, departmental allocation, maintenance or warranty status, and functional condition. This design was considered appropriate for evaluating real-world maintenance patterns without experimental intervention. The hospital setting included high-dependency clinical areas in which equipment availability directly affects operative scheduling, diagnostic timeliness, patient monitoring, and continuity of tertiary care services.

Data source and study population

The data were obtained from the hospital’s official biomedical equipment inventory maintained for equipment monitoring and maintenance oversight. The dataset contained 458 equipment entries from Pediatric Surgery, Laboratory Medicine, Urology, Obstetrics and Gynaecology, and the Dental Institute. Neurosurgery was identified in the source inventory but was excluded from equipment-level analysis because complete item-wise data were unavailable. The study population consisted of biomedical equipment recorded in the departmental inventories, and the unit of analysis was each equipment item. Equipment entries were included when the department, equipment identity, maintenance-contract or warranty status, and functional status were sufficiently documented for classification. Duplicate entries, records without identifiable equipment details, and records lacking both maintenance-coverage and functionality information were excluded from the relevant analysis. Records with partial information were retained only for analyses supported by the available variables.

Data quality verification and missing-data handling

Inventory records were reviewed for completeness and internal consistency by checking equipment descriptions, departmental allocation, contract or warranty status, and recorded functional condition. Apparent inconsistencies were reviewed against the available departmental inventory and maintenance documentation. No missing values were imputed. Equipment records with incomplete information were excluded only from calculations requiring the missing variable, and the available denominator was used for each analysis.

Variables and operational definitions

Maintenance coverage status was the primary exposure variable and included AMC, CMC, active warranty, expired coverage, and no active contract. Functionality status, determined from inventory documentation, was the primary outcome variable and was classified as functional or non-functional. For this audit, equipment downtime was operationally represented by documented non-functional status because continuous downtime duration was not available. This measure reflects equipment status at the time of inventory review and does not represent the exact duration or frequency of downtime. Department was used as a stratification variable to assess departmental vulnerability. Equipment age was determined from the recorded purchase or installation date when available. Contract status was evaluated using documented maintenance agreements, warranty-expiry dates, and renewal status for each listed asset. Clinical criticality was assessed descriptively according to the equipment’s role in life support, surgery, diagnosis, sterilisation, monitoring, or other essential clinical services.

Data categorisation and analysis

Equipment was categorised into three maintenance-protection groups. Assets under active CMC or warranty were classified as protected, assets under AMC were classified as partially protected, and assets without active contract coverage and with warranty expired for more than six months were classified as unprotected. Frequencies, percentages, department-wise distributions, and counts of non-functional equipment were used for descriptive analysis. Department-wise maintenance coverage and functionality rates were compared descriptively, and clusters of clinically important non-functional equipment were identified. The term “comparative evaluation” refers to the descriptive comparison of maintenance coverage and functionality patterns across contract categories and departments rather than a direct inferential comparison of AMC and CMC effectiveness. Denominators were based on the number of eligible equipment entries available for each department and variable. No inferential statistical testing was performed because the dataset was derived from an administrative audit, maintenance categories were unevenly distributed, and complete data on downtime duration, repair turnaround time, service-call frequency, and vendor response were unavailable. The findings were therefore interpreted as descriptive associations and not as causal estimates of maintenance-contract effectiveness.

Ethical considerations

This study did not involve human participants, patient records, biological samples, or direct patient contact. The analysis was limited to administrative equipment-status variables, and the ethical risk was minimal. Access to, extraction of, and analysis of the inventory data were authorised at the institutional level.

## Results

Department-wise equipment distribution and contract coverage

A total of 458 equipment entries were reviewed across five analyzable departments. The Dental Institute contributed the largest share of records, with 302 of 458 entries (65.9%), followed by Pediatric Surgery with 52 of 458 entries (11.4%), Urology with 51 of 458 entries (11.1%), Obstetrics and Gynaecology with 33 of 458 entries (7.2%), and Laboratory Medicine with 20 of 458 entries (4.4%). Neurosurgery was included in the source inventory, but equipment-level data were not available for analysis. AMC/CMC coverage varied substantially across departments. Laboratory Medicine had the highest active AMC/CMC coverage, with 13 of 20 entries (65.0%) covered, whereas Urology had the lowest coverage, with 1 of 51 entries (2.0%) covered. Pediatric Surgery had 8 of 52 entries (15.4%) under AMC/CMC, Obstetrics and Gynaecology had 16 of 33 entries (48.5%), and the Dental Institute had 15 of 302 entries (5.0%). Overall, 53 of 458 equipment entries (11.6%) were under AMC/CMC, whereas 405 of 458 entries (88.4%) had no active contract coverage. Non-functional equipment was recorded in 20 of 458 entries (4.4%), with the highest burden in Urology. Table 1 shows department-wise equipment distribution, AMC/CMC coverage, no active contract status, and non-functional equipment burden across the hospital inventory.

**Table 1:** Department-Wise Contract and Functionality Status (N = 458) Department-wise percentages use each department’s equipment count as the denominator. AMC: Annual Maintenance Contract, CMC: Comprehensive Maintenance Contract

Department	Total Equipment, n/N (%)	Under AMC/CMC, n/N (%)	No Active Contract, n/N (%)	Non-functional Equipment, n/N (%)
Pediatric Surgery	52/458 (11.4)	8/52 (15.4)	44/52 (84.6)	0/52 (0.0)
Laboratory Medicine	20/458 (4.4)	13/20 (65.0)	7/20 (35.0)	1/20 (5.0)
Urology	51/458 (11.1)	1/51 (2.0)	50/51 (98.0)	19/51 (37.3)
Obstetrics and Gynaecology	33/458 (7.2)	16/33 (48.5)	17/33 (51.5)	0/33 (0.0)
Dental Institute	302/458 (65.9)	15/302 (5.0)	287/302 (95.0)	0/302 (0.0)
Total	458/458 (100.0)	53/458 (11.6)	405/458 (88.4)	20/458 (4.4)

Functional status across departments

**Figure 1:**
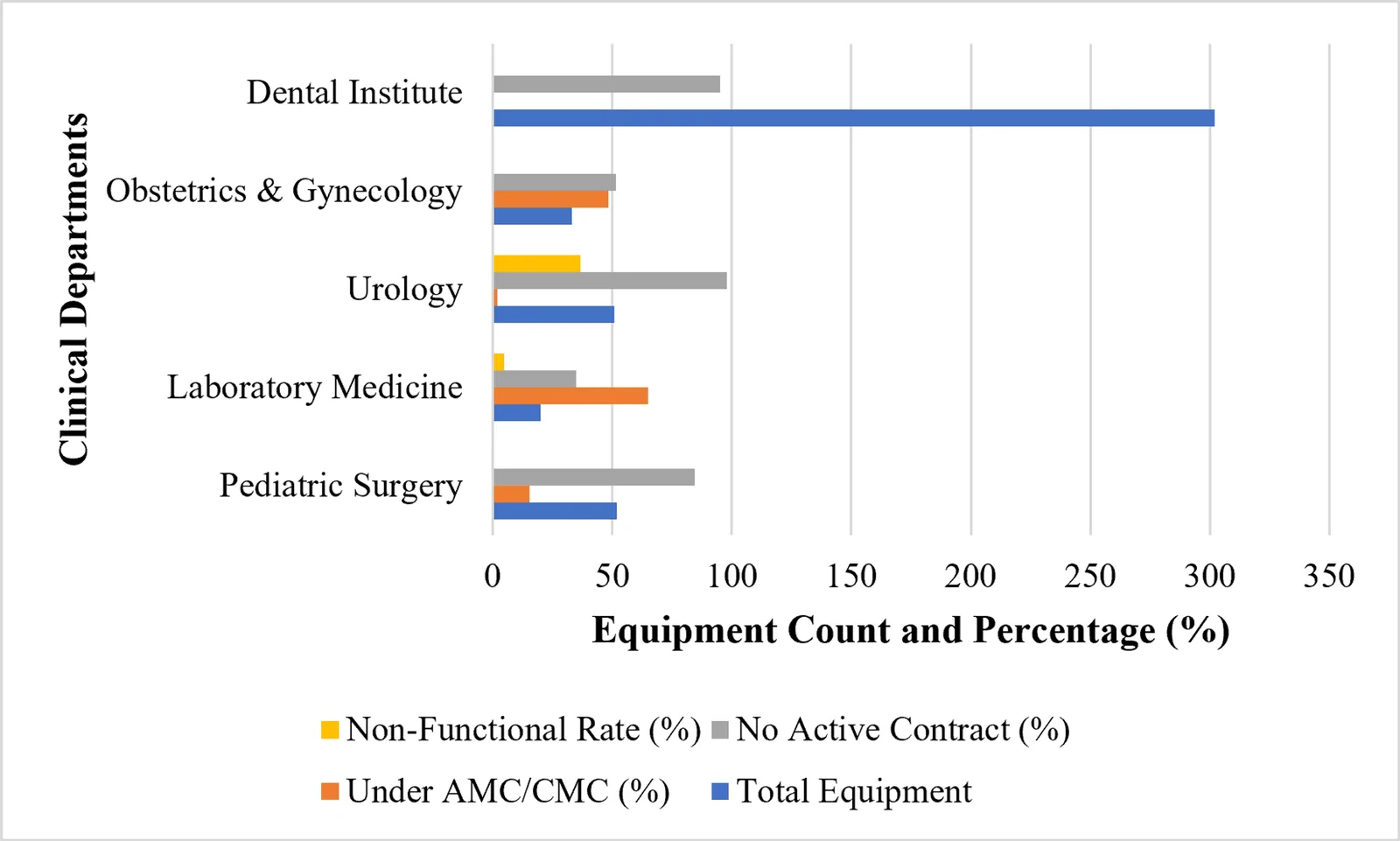
Department-Wise Equipment and Maintenance Status (N = 458) AMC: Annual Maintenance Contract, CMC: Comprehensive Maintenance Contract

Of 458 equipment entries recorded, 20 entries (20/458, 4.4%) were documented as non-functional. Urology accounted for 19 of these 20 non-functional assets (19/20, 95.0%) and 19 of 51 Urology entries (37.3%). Laboratory Medicine accounted for one non-functional item, representing 1 of 20 Laboratory Medicine entries (5.0%). No non-functional equipment was reported in Pediatric Surgery, Obstetrics and Gynaecology, or the Dental Institute, with each department recording 0 entries (0.0%). This distribution shows that equipment non-functionality was not evenly distributed across departments but was concentrated in one high-risk clinical service area with minimal active maintenance-contract coverage. Figure 1 depicts the distribution of equipment, AMC/CMC coverage, no active contracts and non-functional equipment rates by department, with Urology having the highest burden in maintenance and non-functional equipment.

Departmental vulnerability associated with absent contract coverage

Urology showed the highest departmental vulnerability, with 50 of 51 equipment entries (98.0%) having no active AMC/CMC coverage and 19 of 51 entries (37.3%) recorded as non-functional. The Dental Institute also had high no-contract coverage, with 287 of 302 entries (95.0%) lacking active AMC/CMC coverage, although 0 of 302 entries (0.0%) were documented as non-functional. Pediatric Surgery had 44 of 52 entries (84.6%) without active AMC/CMC coverage and 0 of 52 entries (0.0%) recorded as non-functional. Obstetrics and Gynaecology had 17 of 33 entries (51.5%) without active AMC/CMC coverage and 0 of 33 entries (0.0%) recorded as non-functional. Laboratory Medicine had 7 of 20 entries (35.0%) without active AMC/CMC coverage and 1 of 20 entries (5.0%) recorded as non-functional. These findings indicate that absent maintenance-contract coverage may increase equipment vulnerability, particularly when the equipment is clinically critical, high-value, procedure-dependent, or difficult to repair rapidly. Table 2 ranks departments according to absence of active maintenance-contract coverage and corresponding non-functional equipment burden.

**Table 2:** Departments Ranked by Absence of Active Contract Coverage (N = 458) Percentages use department-wise denominators.

Rank	Department	No Active Contract, n/N (%)	Non-functional Equipment, n/N (%)
1	Urology	50/51 (98.0)	19/51 (37.3)
2	Dental Institute	287/302 (95.0)	0/302 (0.0)
3	Pediatric Surgery	44/52 (84.6)	0/52 (0.0)
4	Obstetrics and Gynaecology	17/33 (51.5)	0/33 (0.0)
5	Laboratory Medicine	7/20 (35.0)	1/20 (5.0)

High-risk non-functional equipment cluster

The non-functional equipment cluster in Urology contained high-value and procedure-dependent equipment such as an anaesthesia machine, C-arm machine, cystoscope set, washer disinfector, and surgical diathermy unit. Some of these assets were still under warranty, but were not covered by a current CMC when inventory took place. The clinical significance of this bundle is significant, covering the delivery of anaesthesia, fluoroscopic guidance, endourological treatment, sterilisation process and dissection of the tissue during operation. Clustering of non-functional equipment in one procedural department suggests that there is a service continuity risk in that area that could be a priority area for corrective maintenance and contract review. Selected high-risk non-functional equipment in Urology, along with the recorded status, clinical criticality and probable maintenance gaps, is shown in Table 3.

**Table 3:** Selected Non-functional Equipment in Urology (Total N = 458; Urology N = 51) CMC: Comprehensive Maintenance Contract

Equipment	Clinical Criticality	Recorded Status	Probable Maintenance Gap
Anesthesia Machine	High	Not working	Warranty expired; no active CMC
C-arm Machine	High	Not functional	No active contract
Cystoscope Set	High	Not functional	Warranty expired; no active contract
Washer Disinfector	Moderate	Not functional	Warranty expired; no active contract
Surgical Diathermy	Moderate	Not functional	Warranty expired; no active contract

Relationship between contract coverage and functionality

**Figure 2:**
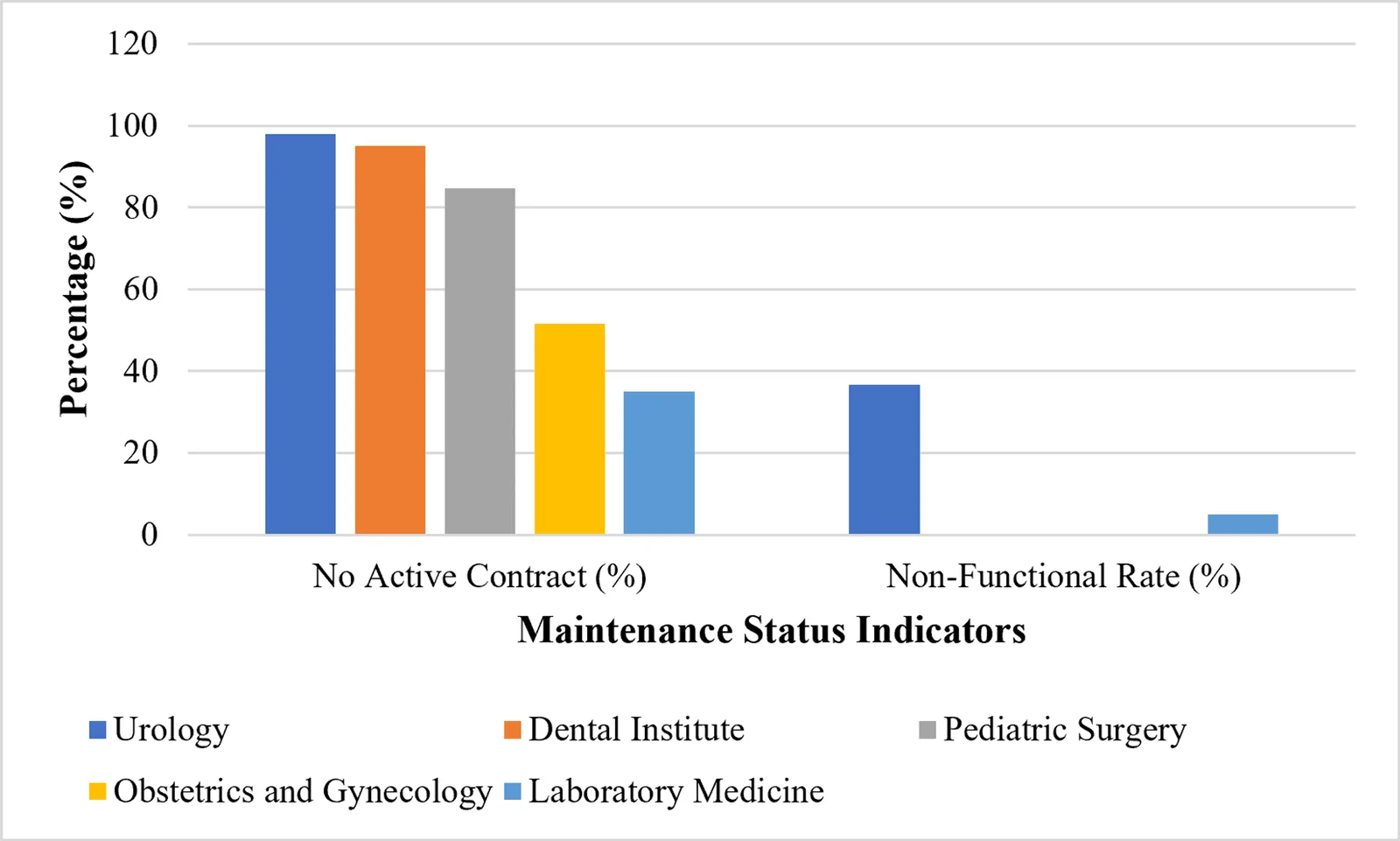
Department-Wise No Active Contract Coverage and Non-functional Equipment Rate (N = 458) Percentages use department-wise denominators.

The association between absent AMC/CMC coverage and non-functional equipment burden was most pronounced in Urology, where 50 of 51 equipment entries (98.0%) had no active contract, and 19 of 51 entries (37.3%) were non-functional. Laboratory Medicine had comparatively higher AMC/CMC coverage, with 13 of 20 entries (65.0%) under active coverage, and only 1 of 20 entries (5.0%) was recorded as non-functional. Pediatric Surgery had 44 of 52 entries (84.6%) without active AMC/CMC coverage and 0 of 52 entries (0.0%) recorded as non-functional, whereas the Dental Institute had 287 of 302 entries (95.0%) without active AMC/CMC coverage and 0 of 302 entries (0.0%) recorded as non-functional. Obstetrics and Gynaecology had 17 of 33 entries (51.5%) without active AMC/CMC coverage and 0 of 33 entries (0.0%) recorded as non-functional. These findings indicate that contract status alone does not fully explain functionality, as equipment criticality, age, utilisation, repair complexity, backup availability, and documentation completeness may also influence recorded non-functionality. Figure 2 presents department-wise no active contract coverage and non-functional equipment rates, showing Urology as the most vulnerable department.

The audit results revealed three main findings. First, a major institutional maintenance-planning gap was identified, as 405 of 458 equipment entries (88.4%) had no active AMC/CMC coverage. Second, non-functionality was highly concentrated in Urology, where 19 of 51 entries (37.3%) were non-functional, and 50 of 51 entries (98.0%) lacked active AMC/CMC coverage. Third, high no-contract coverage was not uniformly associated with documented non-functionality across all departments, as Pediatric Surgery had 44 of 52 entries (84.6%) without active coverage and the Dental Institute had 287 of 302 entries (95.0%) without active coverage, yet both departments recorded 0 non-functional entries (0.0%). Contract status should therefore be interpreted alongside equipment criticality, age, utilisation intensity, and in-house repair capacity.

## Discussion

The findings indicate an association between equipment non-functionality, continuity of maintenance coverage, clinical criticality, and departmental maintenance governance in the tertiary care setting. Equipment without active maintenance coverage may be more susceptible to prolonged downtime, particularly when devices are procedure-dependent, high-cost, or difficult to repair through routine in-house processes. Concentration of non-functional equipment within a procedural department may reflect weaknesses in contract renewal, spare-part procurement, vendor coordination, or internal escalation processes, converting a technical maintenance issue into a clinical service-continuity concern. Such downtime may delay procedures, reduce departmental capacity, increase referrals or outsourcing, generate emergency repair expenditure, and place avoidable pressure on hospital budgets. Hospital operations teams, physician leaders, biomedical engineers, and financial administrators should therefore jointly review the maintenance status of clinically critical equipment. Equipment functionality was not determined by contract status alone, as some departments with limited formal coverage recorded no non-functional assets. Equipment age, technical complexity, utilisation intensity, availability of backup devices, local repair capacity, and documentation completeness may also influence functionality. These findings support risk-based maintenance planning rather than uniform contract allocation, with decisions guided by clinical consequences, service dependency, repair complexity, and the anticipated impact of downtime.

The clinical distinction between AMC and CMC was also relevant to the audit findings. AMC generally provides scheduled preventive maintenance and breakdown service support, but restoration may be delayed when major spare parts are excluded and require separate approval or procurement. CMC provides broader protection by combining preventive maintenance, corrective repair, labour, travel, and spare-part coverage within a single agreement. This model may be more appropriate for high-value, high-utilisation, and clinically critical devices whose failure could interrupt surgery, anaesthesia care, diagnostic services, sterilisation processes, or patient monitoring. Moderate-risk equipment may be managed through AMC when clear spare-part procurement and escalation pathways are available. Low-risk, inexpensive, or readily replaceable equipment may be maintained through structured in-house biomedical engineering support or pay-per-repair arrangements.

The implication is that maintenance contracts must be viewed as a means of patient-safety governance and clinical service continuity, rather than just a cost item. Equipment that does not function in specific areas such as surgery or diagnosis may result in delays in procedures, extended patient wait times, reduced operating room efficiency and reliance on referrals or other equipment [12]. This disruption can have an impact on the outcome of the patient and also the efficiency of the hospital in tertiary care hospitals [13]. There is a need for a three-stage maintenance system. Comprehensive support, using CMC or equivalent, should be provided to critical and high-utilisation equipment. Some equipment that is considered moderate-risk can be handled through AMC, provided pre-approved spare-part pathways exist [14]. Equipment that is not expensive, critical, or readily replaceable can have in-house biomedical maintenance or be serviced by a local vendor [15]. This stratification can help minimise unnecessary downtime without expensive all-inclusive contracts for all assets. The results also confirm the need for a centralised equipment-management system. Routine biomedical governance should include warranty-expiry alerts, service-report tracking, downtime register, vendor-response monitoring, and periodic functionality audits. Such a strategy is useful for preparedness for accreditation, accountability, and decision-making for capital replacement and maintenance budgeting.

The results agree with other hospital-based assessments that equipment in CMC typically has higher availability than equipment that is not maintained under comprehensive contracting arrangements [16]. Previous reports have also highlighted the importance of preventive maintenance, rapid response from vendors and availability of spare parts for equipment uptime in clinical settings [17]. The vulnerability of equipment that is not on an active contract is consistent with previous findings that reactive maintenance strategies have been linked to late repairs, extended equipment downtime, and decreased operational reliability [18]. In the same vein, it has been reported in other studies that warranties or comprehensive service models can be a close approximation to the manufacturer's level of support in the case of high-dollar diagnostic and therapeutic machines [19].

The results that some departments had working equipment even if the contracts were not fully covered are consistent with literature that shows that in-house biomedical capacity, equipment type, age and criticality can affect the impact of contract status [20]. This facilitates selective maintenance, which is suitable for critical and high-cost items where CMC needs to be implemented, and for other equipment for which there is less urgency for action, structured in-house or AMC systems can be used. In general, the results of the present study strengthen the current evidence by incorporating the relationship between contract status and departmental functional patterns in an inventory of a tertiary care hospital. The results support a practical model in which biomedical equipment maintenance is planned according to clinical risk, service dependency, contract coverage, and documented equipment performance.

Limitations and future recommendations

This study has several limitations. The analysis used secondary biomedical equipment inventory data from a single tertiary care hospital, which may limit the generalisability of the findings to other institutions. Functionality status was used as a proxy for equipment downtime because exact downtime duration, repair turnaround time, service-call frequency, vendor response time, and repeated failure data were unavailable. This approach identifies equipment recorded as non-functional but does not capture the duration, frequency, or full operational impact of equipment unavailability. Neurosurgery records were incomplete, and some warranty or service-report entries may also have been incomplete or inaccurately documented. The descriptive audit design does not establish a causal relationship between maintenance-contract status and equipment functionality. Equipment age, utilisation intensity, technical complexity, spare-part availability, backup equipment, vendor performance, and in-house biomedical engineering capacity may also have influenced functional status.

Future studies should use prospective, multicentre designs with standardised definitions of downtime, computerised maintenance-management systems, and real-time repair tracking. Risk models should incorporate equipment criticality, age, utilisation, spare-part cost and availability, vendor response time, repair turnaround time, and institutional biomedical engineering capacity. Automated warranty-expiry alerts, department-wise downtime registers, periodic service-report audits, and tiered maintenance-contract strategies should be adopted. CMC or equivalent comprehensive support should be prioritised for clinically critical, high-value, and high-utilisation equipment, whereas AMC, structured in-house maintenance, or pay-per-repair arrangements may be suitable for lower-risk assets.

## Conclusions

The present study highlights that continuity of maintenance contracts, criticality of the equipment and departmental governance of maintenance have a significant impact on medical equipment downtimes in a tertiary care hospital. An absence of active AMC/CMC coverage was identified as a significant weak point and was particularly identified as a vulnerability in clinically critical and high-value equipment used in procedural services. CMC may be more appropriate for life-support, operative, diagnostic, and high-utilisation equipment because it provides broader maintenance coverage, including corrective repair and spare-part support. The present findings indicate an association between limited maintenance coverage and equipment non-functionality but do not establish that CMC directly reduces downtime. In moderate-risk equipment, good biomedical oversight coupled with timely spare procurement may allow AMC to be considered as appropriate. Low-risk, easy-to-replace equipment can be handled using a system of either structured in-house equipment management or pay-per-repair. The results are in favour of a tiered approach to maintenance instead of equal allocation of contracts by risk. Warranty tracking, downtime registers, service report audits and computerised maintenance systems can help support clinical service continuity, enhance accreditation readiness and minimise unnecessary functional loss. Maintenance contracts should be considered a component of patient safety and service continuity in hospital quality-management systems.
